# Diallyl 5-[(4-hexyl­oxyphen­yl)imino­meth­yl]-*m*-phenyl­ene dicarbonate

**DOI:** 10.1107/S1600536809041142

**Published:** 2009-10-23

**Authors:** Ana María Herrera-González, Delia López-Velázquez, Sylvain Bernès

**Affiliations:** a Centro de Investigaciones en Materiales y Metalurgia, Universidad Autónoma del estado de Hidalgo, Carretera Pachuca Tulancingo Km 4.5, 42184 Mineral de la Reforma, Hgo., Mexico; b Facultad de Ciencias Químicas, Universidad Autónoma de Puebla, Boulevard 14 Sur, Col. San Manuel, 72570 Puebla, Pue., Mexico; c DEP Facultad de Ciencias Químicas, UANL, Guerrero y Progreso S/N, Col. Treviño, 64570 Monterrey, N.L., Mexico

## Abstract

The title mol­ecule, C_27_H_31_NO_7_, an imine derivative bearing both carbonate and allyl functionalities, was synthesized in the hope of obtaining a mesogenic polymerizable material. The allyl­carbonate arms are fully disordered over two sets of sites, reflecting a large degree of rotational freedom about σ bonds [occupancies: 0.665 (9)/0.335 (9) for one substituent, 0.564 (9)/0.436 (9) for the other]. In contrast, the hexyl chain is ordered, and presents the common all-*trans* extended conformation. The benzene rings connected *via* the imine group make a dihedral angle of 9.64 (11)°. In the crystal, the Y-shaped mol­ecules are weakly associated into centrosymmetric dimers through pairs of C—H⋯O(hex­yl) contacts. The resulting layers of dimers, approximately parallel to (2
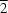
5), are closely packed in the crystal, allowing π⋯π inter­actions between benzene rings of neighboring layers: the separation between the centroid of the benzene ring substituted by allyl­carbonate and the centroid of the benzene ring bearing the hex­yloxy group in the adjacent layer is 3.895 (1) Å.

## Related literature

For the crystal structure of 4-(hex­yloxy)aniline, used as a starting material, see: Herrera *et al.* (2005 ▶). For the crystal structures of mol­ecules with allycarbonate functionality, see: Michelet *et al.* (2003 ▶); Burns & Forsyth (2008 ▶); Flores Ahuactzin *et al.* (2009 ▶). For applications of the above mol­ecules as polymerizable monomers, see: Herrera (2006 ▶).
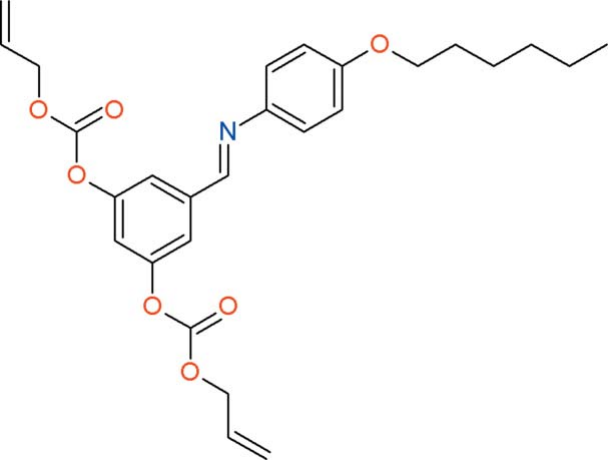


## Experimental

### 

#### Crystal data

C_27_H_31_NO_7_
*M* *_r_* = 481.53
Triclinic, 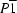

*a* = 8.6407 (11) Å
*b* = 10.9711 (14) Å
*c* = 15.014 (2) Å
α = 102.756 (11)°
β = 103.368 (12)°
γ = 101.092 (12)°
*V* = 1305.4 (3) Å^3^
*Z* = 2
Mo *K*α radiation
μ = 0.09 mm^−1^
*T* = 298 K
0.6 × 0.6 × 0.2 mm

#### Data collection

Bruker P4 diffractometer
Absorption correction: none
8105 measured reflections
5927 independent reflections
3505 reflections with *I* > 2σ(*I*)
*R*_int_ = 0.027
3 standard reflections every 97 reflections intensity decay: 1%

#### Refinement

*R*[*F* ^2^ > 2σ(*F* ^2^)] = 0.053
*wR*(*F* ^2^) = 0.165
*S* = 1.04
5927 reflections
409 parameters
20 restraints
H-atom parameters constrained
Δρ_max_ = 0.14 e Å^−3^
Δρ_min_ = −0.15 e Å^−3^

### 

Data collection: *XSCANS* (Siemens, 1996 ▶); cell refinement: *XSCANS*; data reduction: *XSCANS*; program(s) used to solve structure: *SHELXS97* (Sheldrick, 2008 ▶); program(s) used to refine structure: *SHELXL97* (Sheldrick, 2008 ▶); molecular graphics: *Mercury* (Macrae *et al.*, 2006 ▶); software used to prepare material for publication: *SHELXL97*.

## Supplementary Material

Crystal structure: contains datablocks I, global. DOI: 10.1107/S1600536809041142/ci2922sup1.cif

Structure factors: contains datablocks I. DOI: 10.1107/S1600536809041142/ci2922Isup2.hkl

Additional supplementary materials:  crystallographic information; 3D view; checkCIF report

## Figures and Tables

**Table 1:** Hydrogen-bond geometry (Å, °)

*D*—H⋯*A*	*D*—H	H⋯*A*	*D*⋯*A*	*D*—H⋯*A*
C13—H13*A*⋯O15^i^	0.93	2.60	3.511 (2)	166

Symmetry code: (i) 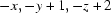.

**Fig. 1.**
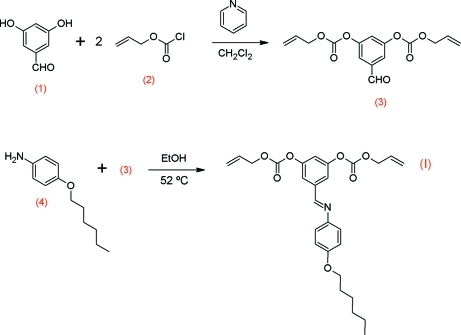
The synthetic route used for the preparation of (I).

**Fig. 2.**
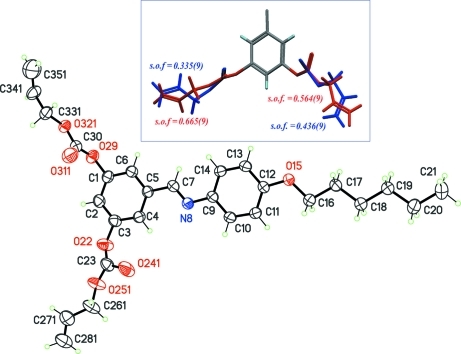
The molecular structure of (I), with displacement ellipsoids shown at the 30% probability level. Only major disorder components are shown. The inset represents the central benzene ring with disordered allylcarbonate groups: the major disorder component is shown in red and the minor component in blue; site occupation factors are quoted.

**Fig. 3.**
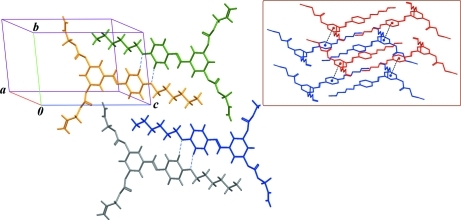
A part of the crystal structure of (I), with C—H···O contacts represented as dashed lines. The four represented molecules lie in a layer, and colors scheme is related to symmetry codes; green: -x, 1 - y, 2 - z; gold: asymmetric unit; blue: -1 - x, -y, 2 - z; grey: -1 + x, -1 + y, z. The inset represents two layers (red and blue) with π···π interactions represented as dashed lines.

## supplementary crystallographic information

### Comment

We are involved in a general project dealing with the synthesis of new allyl carbonate compounds, with the hope to obtain suitable monomers for the preparation of glasses with designed properties (Herrera, 2006). The previous report in this series was about allyl 4-hydroxyphenyl carbonate (Flores Ahuactzin *et al.*, 2009), which was found to display a disordered allyl functionality in the solid-state.

The title molecule, (I), is based on a benzene core di-substituted by allyl carbonate groups. The molecule is completed by a third substituent, derived from 4-(hexyloxy)aniline, for which the X-ray structure has been also reported (Herrera *et al.*, 2005). The whole molecule is a Schiff base including chemical features expected to give a mesogenic behavior to the material. This compound can be prepared following two routes, starting from 3,5-dihydroxybenzaldehyde. The two steps route (Fig. 1 and *experimental*) consists of the functionalization of 3,5-dihydroxybenzaldehyde using allylchloroformate, followed by condensation with 4-(hexyloxy)aniline, to form the Schiff base. The alternative route (Herrera, 2006) is to prepare the Schiff base prior to functionalize with allylchloroformate.

The resulting compound (Fig. 2) is characterized by strongly disordered allylcarbonate substituents: five of the seven atoms in each substituent are disordered over two sites (Fig. 2, inset), with occupancies being 0.564 (9) and 0.436 (9) for one arm, and 0.665 (9) and 0.335 (9) in the other. A remarkable arrangement is observed in the first substituent (O22···C28), where disordered final CCH_2_ groups are placed almost perpendicular, reflecting a high degree of free rotation about σ bonds in these substituents. This behavior, resulting in a variety of stable conformations for the allylcarbonate functional groups, has been also observed in related structures (Michelet *et al.*, 2003; Burns & Forsyth, 2008; Flores Ahuactzin *et al.*, 2009). In (I), the observed disorder may be related to the rather low melting point of this material, 318 K (45° C).

The imine component is ordered, and the hexyl chain presents the common all-*trans* conformation. The complete molecule is Y-shaped, with a small dihedral angle of 9.64 (11)° between the benzene rings.

The crystal structure (Fig. 3) contains centrosymmetric dimers, formed through weak *C*—*H*···*O*(hexyl) contacts. Dimers are arranged in planes, approximately parallel to the (225) in the crystal. Two neighboring layers are in close contact *via*π···π interactions between benzene rings (Fig. 3, inset). The centroid of the benzene ring substituted by allylcarbonate and the centroid of the benzene ring bearing the hexyloxy group in the following layer (symmetry code: *x* - 1, *y*, *z*), are separated by 3.895 (1) Å.

### Experimental

A solution of 3,5-dihydroxybenzaldehyde (0.5 g, 3.6 mmol) in CH_2_Cl_2_ and pyridine as catalyst was cooled in an ice bath, and allylchloroformate (0.95 g, 7.8 mmol) was added dropwise under stirring at 278 K. The mixture was stirred for 4 h under an atmosphere of Ar. The reaction was then treated with a solution of HCl at 5%, and concentrated under reduced pressure, yielding the crude aldehyde (3) as a liquid (Fig. 1). This intermediate was purified by column chromatography on silica, eluting with CH_2_Cl_2_. Yield 91%. To a solution of (3) (0.5 g, 1.6 mmol) in dry ethanol (30 ml) was added 4-(hexyloxy)aniline (0.33 g, 1.6 mmol) under an atmosphere of Ar. The reaction mixture was then heated to 325 K for 18 h. The reaction mixture was cooled to room temperature and solvent eliminated under reduced pressure, affording (I), which was purified by column chromatography on silica, eluting with CH_2_Cl_2_ and then recrystallized from methanol (93% yield; brown crystals).

### Refinement

Both allycarbonate groups are disordered over two positions. Atoms O24, O25, C26, C27 and C28 are disordered over two sites (O241/O251/C261/C271/C281 and O242/O252/C262/C272/C282), with refined occupancies of 0.564 (9) and 0.436 (9). In the same way, atoms O31, O32, C33, C34 and C35 are disordered over two positions (O311/O321/C331/C341/C351 and O312/O322/C332/C342/C352), with occupancies of 0.665 (9) and 0.335 (9). Bond lengths in these disordered fragments were restrained using target values afforded by *Mogul*; for the first disordered part, restraints are as follows: C23—O241 = 1.21 (1) Å; C23—O251 = 1.33 (1) Å; O251—C261 = 1.46 (1) Å; C261—C271 = 1.47 (1) Å; C271—C281 = 1.27 (1) Å. Similar restraints were used in other disordered parts. All H atoms were placed in calculated positions and refined as riding to their parent atoms. C—H bond lengths were fixed to 0.96 (methyl), 0.97 (methylene), or 0.93 Å (all other H toms). Isotropic displacement parameters for H atoms were calculated as *U*_iso_(H) = 1.2*U*_eq_(carrier atom), except for methyl C21, for which *U*_iso_(H) = 1.5*U*_eq_(C21).

### Crystal data

**Table:**

C_27_H_31_NO_7_	*Z* = 2
*M**_r_* = 481.53	*F*(000) = 512
Triclinic, *P*1	*D*_x_ = 1.225 Mg m^−^^3^
Hall symbol: -P 1	Melting point: 318 K
*a* = 8.6407 (11) Å	Mo *K*α radiation, λ = 0.71073 Å
*b* = 10.9711 (14) Å	Cell parameters from 70 reflections
*c* = 15.014 (2) Å	θ = 4.5–12.5°
α = 102.756 (11)°	µ = 0.09 mm^−^^1^
β = 103.368 (12)°	*T* = 298 K
γ = 101.092 (12)°	Plate, brown
*V* = 1305.4 (3) Å^3^	0.6 × 0.6 × 0.2 mm

### Data collection

**Table:**

Bruker P4 diffractometer	*R*_int_ = 0.027
Radiation source: fine-focus sealed tube	θ_max_ = 27.5°, θ_min_ = 2.0°
graphite	*h* = −11→3
2θ/ω scans	*k* = −13→13
8105 measured reflections	*l* = −19→19
5927 independent reflections	3 standard reflections every 97 reflections
3505 reflections with *I* > 2σ(*I*)	intensity decay: 1%

### Refinement

**Table:**

Refinement on *F*^2^	Primary atom site location: structure-invariant direct methods
Least-squares matrix: full	Secondary atom site location: difference Fourier map
*R*[*F*^2^ > 2σ(*F*^2^)] = 0.053	Hydrogen site location: inferred from neighbouring sites
*wR*(*F*^2^) = 0.165	H-atom parameters constrained
*S* = 1.04	*w* = 1/[σ^2^(*F*_o_^2^) + (0.0692*P*)^2^ + 0.1545*P*] where *P* = (*F*_o_^2^ + 2*F*_c_^2^)/3
5927 reflections	(Δ/σ)_max_ = 0.001
409 parameters	Δρ_max_ = 0.14 e Å^−^^3^
20 restraints	Δρ_min_ = −0.15 e Å^−^^3^
0 constraints	

### Fractional atomic coordinates and isotropic or equivalent isotropic displacement parameters (Å^2^)

**Table:**

	*x*	*y*	*z*	*U*_iso_*/*U*_eq_	Occ. (<1)
C1	0.8795 (2)	0.36570 (18)	0.72744 (13)	0.0647 (4)	
C2	0.8794 (2)	0.2444 (2)	0.67888 (14)	0.0715 (5)	
H2A	0.9542	0.2323	0.6442	0.086*	
C3	0.7648 (2)	0.14046 (19)	0.68288 (15)	0.0704 (5)	
C4	0.6528 (2)	0.15553 (18)	0.73416 (14)	0.0648 (4)	
H4A	0.5769	0.0841	0.7361	0.078*	
C5	0.6560 (2)	0.28065 (17)	0.78313 (12)	0.0602 (4)	
C6	0.7713 (2)	0.38578 (18)	0.78032 (13)	0.0645 (4)	
H6A	0.7756	0.4691	0.8138	0.077*	
C7	0.5327 (2)	0.30287 (18)	0.83354 (13)	0.0646 (4)	
H7A	0.5447	0.3860	0.8706	0.078*	
N8	0.41161 (18)	0.21453 (14)	0.82888 (11)	0.0642 (4)	
C9	0.2901 (2)	0.24258 (16)	0.87404 (12)	0.0593 (4)	
C10	0.1795 (2)	0.13913 (17)	0.88151 (14)	0.0692 (5)	
H10A	0.1877	0.0558	0.8569	0.083*	
C11	0.0566 (2)	0.15580 (17)	0.92465 (14)	0.0706 (5)	
H11A	−0.0160	0.0845	0.9292	0.085*	
C12	0.0425 (2)	0.27938 (16)	0.96082 (12)	0.0606 (4)	
C13	0.1498 (2)	0.38393 (17)	0.95118 (14)	0.0667 (5)	
H13A	0.1394	0.4671	0.9739	0.080*	
C14	0.2712 (2)	0.36611 (17)	0.90851 (14)	0.0666 (5)	
H14A	0.3419	0.4374	0.9025	0.080*	
O15	−0.07085 (16)	0.30948 (12)	1.00565 (10)	0.0730 (4)	
C16	−0.1880 (2)	0.20872 (17)	1.01915 (14)	0.0683 (5)	
H16A	−0.1319	0.1589	1.0554	0.082*	
H16B	−0.2565	0.1509	0.9581	0.082*	
C17	−0.2914 (2)	0.27331 (17)	1.07262 (13)	0.0666 (5)	
H17A	−0.3449	0.3231	1.0352	0.080*	
H17B	−0.2195	0.3333	1.1320	0.080*	
C18	−0.4212 (2)	0.18124 (18)	1.09486 (16)	0.0740 (5)	
H18A	−0.3678	0.1338	1.1346	0.089*	
H18B	−0.4912	0.1192	1.0359	0.089*	
C19	−0.5273 (2)	0.24900 (19)	1.14540 (15)	0.0734 (5)	
H19A	−0.4567	0.3108	1.2043	0.088*	
H19B	−0.5790	0.2972	1.1057	0.088*	
C20	−0.6588 (3)	0.1610 (2)	1.1682 (2)	0.0974 (7)	
H20A	−0.6073	0.1144	1.2093	0.117*	
H20B	−0.7283	0.0979	1.1095	0.117*	
C21	−0.7661 (3)	0.2299 (3)	1.2164 (2)	0.1085 (8)	
H21A	−0.8454	0.1681	1.2302	0.163*	
H21B	−0.8222	0.2728	1.1749	0.163*	
H21C	−0.6986	0.2925	1.2747	0.163*	
O22	0.77632 (17)	0.01980 (14)	0.63440 (13)	0.1008 (5)	
C23	0.6461 (3)	−0.0735 (2)	0.58199 (16)	0.0801 (6)	
O241	0.5208 (7)	−0.0956 (6)	0.6034 (6)	0.149 (3)	0.564 (9)
O251	0.6943 (11)	−0.1715 (7)	0.5473 (7)	0.102 (3)	0.564 (9)
C261	0.5708 (9)	−0.2733 (8)	0.4693 (6)	0.097 (3)	0.564 (9)
H26A	0.5233	−0.3415	0.4940	0.116*	0.564 (9)
H26B	0.4832	−0.2380	0.4410	0.116*	0.564 (9)
C271	0.6431 (10)	−0.3250 (7)	0.3991 (4)	0.094 (2)	0.564 (9)
H27A	0.6882	−0.2686	0.3684	0.113*	0.564 (9)
C281	0.6523 (11)	−0.4432 (6)	0.3737 (7)	0.116 (2)	0.564 (9)
H28A	0.6088	−0.5031	0.4024	0.140*	0.564 (9)
H28B	0.7024	−0.4683	0.3266	0.140*	0.564 (9)
O242	0.5239 (9)	−0.0401 (7)	0.5506 (6)	0.130 (3)	0.436 (9)
O252	0.6795 (12)	−0.1657 (9)	0.5220 (9)	0.080 (2)	0.436 (9)
C262	0.5589 (18)	−0.2909 (11)	0.4839 (11)	0.122 (5)	0.436 (9)
H26C	0.5454	−0.3263	0.5362	0.147*	0.436 (9)
H26D	0.4537	−0.2787	0.4529	0.147*	0.436 (9)
C272	0.6050 (10)	−0.3823 (10)	0.4167 (7)	0.116 (4)	0.436 (9)
H27B	0.5567	−0.4687	0.4091	0.139*	0.436 (9)
C282	0.7030 (11)	−0.3623 (17)	0.3657 (8)	0.126 (4)	0.436 (9)
H28C	0.7559	−0.2782	0.3695	0.151*	0.436 (9)
H28D	0.7210	−0.4319	0.3249	0.151*	0.436 (9)
O29	0.99760 (15)	0.47296 (13)	0.72656 (10)	0.0782 (4)	
C30	0.9509 (3)	0.5274 (2)	0.65583 (16)	0.0760 (5)	
O311	0.8370 (8)	0.4836 (9)	0.5878 (5)	0.125 (3)	0.665 (9)
O321	1.0645 (7)	0.6334 (6)	0.6683 (5)	0.0712 (12)	0.665 (9)
C331	1.0261 (6)	0.6997 (5)	0.5963 (3)	0.0784 (13)	0.665 (9)
H33A	0.9295	0.7313	0.5994	0.094*	0.665 (9)
H33B	1.0043	0.6416	0.5333	0.094*	0.665 (9)
C341	1.1697 (5)	0.8080 (5)	0.6154 (4)	0.0931 (16)	0.665 (9)
H34A	1.2729	0.7915	0.6231	0.112*	0.665 (9)
C351	1.1595 (16)	0.9217 (10)	0.6217 (10)	0.176 (5)	0.665 (9)
H35A	1.0575	0.9404	0.6142	0.212*	0.665 (9)
H35B	1.2538	0.9874	0.6339	0.212*	0.665 (9)
O312	0.8084 (8)	0.5001 (13)	0.6096 (7)	0.074 (2)	0.335 (9)
O322	1.0978 (15)	0.6155 (15)	0.6718 (12)	0.110 (5)	0.335 (9)
C332	1.122 (2)	0.6920 (11)	0.6036 (9)	0.119 (4)	0.335 (9)
H33C	1.2353	0.7073	0.6024	0.143*	0.335 (9)
H33D	1.0529	0.6423	0.5402	0.143*	0.335 (9)
C342	1.081 (2)	0.8163 (11)	0.6282 (10)	0.173 (9)	0.335 (9)
H34B	0.9841	0.8117	0.6459	0.207*	0.335 (9)
C352	1.1533 (14)	0.9284 (7)	0.6298 (11)	0.086 (4)	0.335 (9)
H35C	1.2511	0.9419	0.6132	0.103*	0.335 (9)
H35D	1.1088	0.9975	0.6476	0.103*	0.335 (9)

### Atomic displacement parameters (Å^2^)

**Table:**

	*U*^11^	*U*^22^	*U*^33^	*U*^12^	*U*^13^	*U*^23^
C1	0.0521 (9)	0.0710 (11)	0.0688 (11)	0.0102 (8)	0.0160 (8)	0.0214 (9)
C2	0.0545 (10)	0.0824 (13)	0.0786 (12)	0.0185 (9)	0.0254 (9)	0.0167 (10)
C3	0.0586 (10)	0.0677 (11)	0.0853 (13)	0.0237 (9)	0.0239 (9)	0.0124 (9)
C4	0.0578 (10)	0.0609 (10)	0.0805 (12)	0.0187 (8)	0.0254 (9)	0.0204 (9)
C5	0.0568 (9)	0.0638 (10)	0.0621 (10)	0.0184 (8)	0.0190 (8)	0.0169 (8)
C6	0.0636 (10)	0.0618 (10)	0.0663 (10)	0.0164 (8)	0.0186 (9)	0.0138 (8)
C7	0.0687 (11)	0.0609 (10)	0.0660 (11)	0.0207 (9)	0.0237 (9)	0.0131 (8)
N8	0.0632 (9)	0.0596 (8)	0.0754 (10)	0.0200 (7)	0.0285 (7)	0.0173 (7)
C9	0.0613 (10)	0.0559 (9)	0.0634 (10)	0.0191 (8)	0.0223 (8)	0.0135 (8)
C10	0.0741 (11)	0.0516 (9)	0.0873 (13)	0.0194 (9)	0.0363 (10)	0.0133 (9)
C11	0.0737 (11)	0.0525 (10)	0.0928 (13)	0.0144 (9)	0.0406 (11)	0.0184 (9)
C12	0.0641 (10)	0.0575 (10)	0.0646 (10)	0.0197 (8)	0.0264 (8)	0.0137 (8)
C13	0.0725 (11)	0.0501 (9)	0.0821 (12)	0.0192 (8)	0.0324 (10)	0.0137 (8)
C14	0.0701 (11)	0.0514 (9)	0.0852 (12)	0.0158 (8)	0.0341 (10)	0.0199 (9)
O15	0.0790 (8)	0.0577 (7)	0.0936 (9)	0.0184 (6)	0.0497 (7)	0.0167 (6)
C16	0.0694 (11)	0.0599 (10)	0.0797 (12)	0.0155 (9)	0.0315 (10)	0.0182 (9)
C17	0.0684 (11)	0.0619 (10)	0.0660 (11)	0.0122 (8)	0.0259 (9)	0.0075 (8)
C18	0.0776 (12)	0.0634 (11)	0.0877 (13)	0.0185 (9)	0.0389 (11)	0.0180 (10)
C19	0.0775 (12)	0.0646 (11)	0.0777 (12)	0.0128 (9)	0.0338 (10)	0.0116 (9)
C20	0.1057 (17)	0.0846 (15)	0.1256 (19)	0.0259 (13)	0.0658 (16)	0.0412 (14)
C21	0.1053 (18)	0.121 (2)	0.122 (2)	0.0332 (16)	0.0684 (16)	0.0377 (17)
O22	0.0640 (8)	0.0730 (9)	0.1537 (15)	0.0224 (7)	0.0389 (9)	−0.0042 (9)
C23	0.0691 (13)	0.0855 (14)	0.0833 (14)	0.0292 (12)	0.0259 (11)	0.0064 (11)
O241	0.098 (3)	0.123 (4)	0.194 (6)	−0.009 (2)	0.094 (4)	−0.043 (3)
O251	0.092 (3)	0.075 (3)	0.110 (6)	0.035 (2)	0.003 (3)	−0.017 (3)
C261	0.072 (4)	0.091 (5)	0.099 (5)	0.030 (4)	0.006 (3)	−0.020 (4)
C271	0.080 (4)	0.110 (5)	0.075 (3)	0.021 (4)	0.005 (3)	0.011 (3)
C281	0.118 (5)	0.098 (4)	0.114 (5)	0.033 (4)	0.030 (4)	−0.009 (4)
O242	0.097 (4)	0.118 (5)	0.129 (5)	0.062 (4)	−0.029 (3)	−0.025 (3)
O252	0.066 (4)	0.093 (4)	0.089 (5)	0.039 (3)	0.031 (4)	0.018 (3)
C262	0.145 (9)	0.091 (7)	0.105 (7)	−0.019 (6)	0.051 (7)	0.001 (6)
C272	0.099 (5)	0.061 (5)	0.186 (9)	0.012 (4)	0.065 (5)	0.013 (5)
C282	0.101 (6)	0.177 (13)	0.118 (6)	0.065 (7)	0.048 (5)	0.036 (8)
O29	0.0621 (7)	0.0825 (9)	0.0840 (9)	0.0036 (7)	0.0179 (7)	0.0274 (7)
C30	0.0905 (16)	0.0677 (12)	0.0688 (13)	0.0123 (11)	0.0321 (12)	0.0140 (10)
O311	0.169 (5)	0.101 (3)	0.071 (3)	−0.008 (3)	0.009 (3)	0.022 (2)
O321	0.0649 (15)	0.070 (2)	0.077 (2)	0.0065 (18)	0.0211 (15)	0.0249 (17)
C331	0.083 (3)	0.078 (2)	0.077 (2)	0.014 (2)	0.024 (2)	0.0321 (18)
C341	0.072 (2)	0.086 (4)	0.137 (4)	0.016 (2)	0.046 (2)	0.050 (3)
C351	0.166 (9)	0.168 (11)	0.214 (12)	0.034 (8)	0.094 (8)	0.058 (8)
O312	0.043 (3)	0.097 (5)	0.060 (4)	−0.009 (2)	−0.009 (3)	0.025 (4)
O322	0.156 (11)	0.089 (5)	0.118 (7)	0.038 (6)	0.080 (7)	0.044 (4)
C332	0.140 (11)	0.120 (9)	0.094 (7)	0.018 (8)	0.050 (8)	0.019 (5)
C342	0.185 (17)	0.26 (3)	0.197 (14)	0.139 (19)	0.122 (13)	0.161 (16)
C352	0.072 (6)	0.038 (4)	0.128 (9)	0.000 (4)	0.004 (6)	0.023 (5)

### Geometric parameters (Å, °)

**Table:**

C1—C2	1.369 (3)	C21—H21C	0.96
C1—C6	1.378 (2)	O22—C23	1.313 (2)
C1—O29	1.406 (2)	C23—O241	1.195 (4)
C2—C3	1.381 (3)	C23—O242	1.211 (4)
C2—H2A	0.93	C23—O251	1.275 (6)
C3—C4	1.381 (3)	C23—O252	1.322 (7)
C3—O22	1.397 (2)	O251—C261	1.464 (7)
C4—C5	1.399 (2)	C261—C271	1.414 (8)
C4—H4A	0.93	C261—H26A	0.97
C5—C6	1.387 (2)	C261—H26B	0.97
C5—C7	1.469 (2)	C271—C281	1.291 (7)
C6—H6A	0.93	C271—H27A	0.93
C7—N8	1.259 (2)	C281—H28A	0.93
C7—H7A	0.93	C281—H28B	0.93
N8—C9	1.421 (2)	O252—C262	1.458 (9)
C9—C10	1.379 (2)	C262—C272	1.440 (9)
C9—C14	1.395 (2)	C262—H26C	0.97
C10—C11	1.387 (2)	C262—H26D	0.97
C10—H10A	0.93	C272—C282	1.287 (9)
C11—C12	1.384 (2)	C272—H27B	0.93
C11—H11A	0.93	C282—H28C	0.93
C12—O15	1.361 (2)	C282—H28D	0.93
C12—C13	1.387 (2)	O29—C30	1.350 (3)
C13—C14	1.373 (2)	C30—O311	1.174 (5)
C13—H13A	0.93	C30—O312	1.208 (6)
C14—H14A	0.93	C30—O321	1.315 (5)
O15—C16	1.432 (2)	C30—O322	1.374 (9)
C16—C17	1.506 (2)	O321—C331	1.444 (6)
C16—H16A	0.97	C331—C341	1.467 (5)
C16—H16B	0.97	C331—H33A	0.97
C17—C18	1.508 (3)	C331—H33B	0.97
C17—H17A	0.97	C341—C351	1.251 (8)
C17—H17B	0.97	C341—H34A	0.93
C18—C19	1.518 (3)	C351—H35A	0.93
C18—H18A	0.97	C351—H35B	0.93
C18—H18B	0.97	O322—C332	1.484 (9)
C19—C20	1.498 (3)	C332—C342	1.463 (10)
C19—H19A	0.97	C332—H33C	0.97
C19—H19B	0.97	C332—H33D	0.97
C20—C21	1.514 (3)	C342—C352	1.261 (10)
C20—H20A	0.97	C342—H34B	0.93
C20—H20B	0.97	C352—H35C	0.93
C21—H21A	0.96	C352—H35D	0.93
C21—H21B	0.96		
			
C2—C1—C6	121.97 (17)	C20—C21—H21C	109.5
C2—C1—O29	119.21 (16)	H21A—C21—H21C	109.5
C6—C1—O29	118.77 (17)	H21B—C21—H21C	109.5
C1—C2—C3	118.05 (17)	C23—O22—C3	122.43 (15)
C1—C2—H2A	121.0	O241—C23—O251	116.3 (6)
C3—C2—H2A	121.0	O242—C23—O251	129.8 (5)
C2—C3—C4	122.21 (17)	O241—C23—O22	123.4 (3)
C2—C3—O22	114.62 (16)	O242—C23—O22	116.0 (4)
C4—C3—O22	123.13 (18)	O251—C23—O22	108.3 (4)
C3—C4—C5	118.46 (17)	O241—C23—O252	119.5 (5)
C3—C4—H4A	120.8	O242—C23—O252	115.9 (7)
C5—C4—H4A	120.8	O22—C23—O252	113.6 (5)
C6—C5—C4	119.89 (16)	C23—O251—C261	115.8 (7)
C6—C5—C7	119.33 (16)	C271—C261—O251	110.4 (7)
C4—C5—C7	120.70 (16)	C271—C261—H26A	109.6
C1—C6—C5	119.40 (17)	O251—C261—H26A	109.6
C1—C6—H6A	120.3	C271—C261—H26B	109.6
C5—C6—H6A	120.3	O251—C261—H26B	109.6
N8—C7—C5	123.01 (17)	H26A—C261—H26B	108.1
N8—C7—H7A	118.5	C281—C271—C261	126.2 (11)
C5—C7—H7A	118.5	C281—C271—H27A	116.9
C7—N8—C9	120.81 (15)	C261—C271—H27A	116.9
C10—C9—C14	117.79 (15)	C271—C281—H28A	120.0
C10—C9—N8	117.22 (15)	C271—C281—H28B	120.0
C14—C9—N8	124.95 (16)	H28A—C281—H28B	120.0
C9—C10—C11	121.88 (16)	C23—O252—C262	117.8 (8)
C9—C10—H10A	119.1	C272—C262—O252	113.0 (10)
C11—C10—H10A	119.1	C272—C262—H26C	109.0
C12—C11—C10	119.52 (16)	O252—C262—H26C	109.0
C12—C11—H11A	120.2	C272—C262—H26D	109.0
C10—C11—H11A	120.2	O252—C262—H26D	109.0
O15—C12—C11	125.58 (16)	H26C—C262—H26D	107.8
O15—C12—C13	115.30 (15)	C282—C272—C262	129.7 (16)
C11—C12—C13	119.12 (15)	C282—C272—H27B	115.1
C14—C13—C12	120.75 (16)	C262—C272—H27B	115.1
C14—C13—H13A	119.6	C272—C282—H28C	120.0
C12—C13—H13A	119.6	C272—C282—H28D	120.0
C13—C14—C9	120.87 (16)	H28C—C282—H28D	120.0
C13—C14—H14A	119.6	C30—O29—C1	115.14 (15)
C9—C14—H14A	119.6	O311—C30—O321	123.3 (5)
C12—O15—C16	119.90 (13)	O312—C30—O321	126.9 (7)
O15—C16—C17	106.86 (14)	O311—C30—O29	127.1 (5)
O15—C16—H16A	110.4	O312—C30—O29	120.5 (6)
C17—C16—H16A	110.4	O321—C30—O29	109.2 (3)
O15—C16—H16B	110.4	O311—C30—O322	131.1 (8)
C17—C16—H16B	110.4	O312—C30—O322	140.5 (9)
H16A—C16—H16B	108.6	O29—C30—O322	98.3 (6)
C16—C17—C18	114.36 (15)	C30—O321—C331	113.6 (4)
C16—C17—H17A	108.7	O321—C331—C341	106.9 (4)
C18—C17—H17A	108.7	O321—C331—H33A	110.3
C16—C17—H17B	108.7	C341—C331—H33A	110.3
C18—C17—H17B	108.7	O321—C331—H33B	110.3
H17A—C17—H17B	107.6	C341—C331—H33B	110.3
C17—C18—C19	113.01 (16)	H33A—C331—H33B	108.6
C17—C18—H18A	109.0	C351—C341—C331	122.8 (9)
C19—C18—H18A	109.0	C351—C341—H34A	118.6
C17—C18—H18B	109.0	C331—C341—H34A	118.6
C19—C18—H18B	109.0	C341—C351—H35A	120.0
H18A—C18—H18B	107.8	C341—C351—H35B	120.0
C20—C19—C18	114.79 (17)	H35A—C351—H35B	120.0
C20—C19—H19A	108.6	C30—O322—C332	120.9 (11)
C18—C19—H19A	108.6	C342—C332—O322	112.6 (11)
C20—C19—H19B	108.6	C342—C332—H33C	109.1
C18—C19—H19B	108.6	O322—C332—H33C	109.1
H19A—C19—H19B	107.5	C342—C332—H33D	109.1
C19—C20—C21	114.1 (2)	O322—C332—H33D	109.1
C19—C20—H20A	108.7	H33C—C332—H33D	107.8
C21—C20—H20A	108.7	C352—C342—C332	131.7 (15)
C19—C20—H20B	108.7	C352—C342—H34B	114.2
C21—C20—H20B	108.7	C332—C342—H34B	114.2
H20A—C20—H20B	107.6	C342—C352—H35C	120.0
C20—C21—H21A	109.5	C342—C352—H35D	120.0
C20—C21—H21B	109.5	H35C—C352—H35D	120.0
H21A—C21—H21B	109.5		
			
C6—C1—C2—C3	0.9 (3)	C4—C3—O22—C23	44.3 (3)
O29—C1—C2—C3	178.39 (17)	C3—O22—C23—O241	−38.6 (7)
C1—C2—C3—C4	−0.3 (3)	C3—O22—C23—O242	24.6 (7)
C1—C2—C3—O22	−177.81 (17)	C3—O22—C23—O251	−179.7 (6)
C2—C3—C4—C5	0.3 (3)	C3—O22—C23—O252	162.6 (7)
O22—C3—C4—C5	177.54 (18)	O241—C23—O251—C261	50.0 (14)
C3—C4—C5—C6	−0.8 (3)	O242—C23—O251—C261	−14.5 (18)
C3—C4—C5—C7	175.94 (17)	O22—C23—O251—C261	−165.8 (8)
C2—C1—C6—C5	−1.4 (3)	O252—C23—O251—C261	−55 (2)
O29—C1—C6—C5	−178.93 (15)	C23—O251—C261—C271	141.0 (8)
C4—C5—C6—C1	1.4 (3)	O251—C261—C271—C281	117.9 (9)
C7—C5—C6—C1	−175.40 (16)	O241—C23—O252—C262	0.7 (17)
C6—C5—C7—N8	170.43 (18)	O242—C23—O252—C262	−61.5 (16)
C4—C5—C7—N8	−6.3 (3)	O251—C23—O252—C262	85 (3)
C5—C7—N8—C9	−175.94 (15)	O22—C23—O252—C262	160.4 (10)
C7—N8—C9—C10	−167.86 (18)	C23—O252—C262—C272	177.8 (12)
C7—N8—C9—C14	14.3 (3)	O252—C262—C272—C282	−23 (2)
C14—C9—C10—C11	−2.2 (3)	C2—C1—O29—C30	91.0 (2)
N8—C9—C10—C11	179.72 (17)	C6—C1—O29—C30	−91.5 (2)
C9—C10—C11—C12	0.5 (3)	C1—O29—C30—O311	−13.5 (6)
C10—C11—C12—O15	−179.23 (18)	C1—O29—C30—O312	13.3 (8)
C10—C11—C12—C13	1.4 (3)	C1—O29—C30—O321	173.9 (4)
O15—C12—C13—C14	178.99 (17)	C1—O29—C30—O322	−174.0 (9)
C11—C12—C13—C14	−1.6 (3)	O311—C30—O321—C331	7.2 (10)
C12—C13—C14—C9	−0.2 (3)	O312—C30—O321—C331	−20.8 (11)
C10—C9—C14—C13	2.1 (3)	O29—C30—O321—C331	−179.8 (5)
N8—C9—C14—C13	179.94 (17)	O322—C30—O321—C331	131 (4)
C11—C12—O15—C16	0.5 (3)	C30—O321—C331—C341	−175.1 (6)
C13—C12—O15—C16	179.83 (16)	O321—C331—C341—C351	−128.8 (9)
C12—O15—C16—C17	178.47 (15)	O311—C30—O322—C332	12 (2)
O15—C16—C17—C18	−179.06 (16)	O312—C30—O322—C332	−18 (3)
C16—C17—C18—C19	−177.82 (17)	O321—C30—O322—C332	−54 (3)
C17—C18—C19—C20	179.53 (19)	O29—C30—O322—C332	171.8 (14)
C18—C19—C20—C21	−178.6 (2)	C30—O322—C332—C342	92 (2)
C2—C3—O22—C23	−138.2 (2)	O322—C332—C342—C352	136.0 (16)

### Hydrogen-bond geometry (Å, °)

**Table:**

*D*—H···*A*	*D*—H	H···*A*	*D*···*A*	*D*—H···*A*
C13—H13A···O15^i^	0.93	2.60	3.511 (2)	166

Symmetry codes: (i) −*x*, −*y*+1, −*z*+2.
